# Splenic Hematoma as a Rare Complication of Colonoscopy; a Case Report 

**Published:** 2020-10-25

**Authors:** Maddalena Zippi, Roberta Pica, Ingrid Febbraro, Francesco Rocco Pugliese, Francesca Liguori

**Affiliations:** 1 Unit of Gastroenterology and Digestive Endoscopy, Sandro Pertini Hospital, Rome, Italy.; 2 Unit of Urgent Digestive Endoscopy, Sandro Pertini Hospital, Rome, Italy.; 3 Emergency Department, Sandro Pertini Hospital, Rome, Italy.

**Keywords:** Colonoscopy, computed tomography, hematoma, spleen, splenic rupture

## Abstract

Splenic injuries after colonoscopy are an uncommon complication, which can lead to potentially unfortunate outcomes. Their management depends on the type of the splenic damage (hematomas, lacerations, rupture). We describe the case of a woman who visited the Emergency Department due to abdominal pain and pre-syncopal condition, which had occurred 12 hours after she underwent a colonoscopy. An abdominal computed tomography scan showed a splenic hematoma and a hemoperitoneum. An emergency splenectomy was performed successfully. Emergency physicians, who are at the forefront of diagnosing and treating patients, should consider this post-endoscopic complication in order to implement a prompt treatment.

## Introduction

Splenic rupture is a rare complication that may occur following colonoscopy. The first description of this condition, due to an endoscopic procedure, dates back to 1974 (1). The main mechanisms involved are: tension of the splenocolic ligament and direct damage due to maneuvers of the colonoscope at the level of splenic flexure (2-4). The most likely predisposing causes are post-surgical adhesions, previous trauma, and underlying splenic pathology (2-4). Hereby, we describe a rare case of a splenic rupture that occurred after lower endoscopy.

## Case presentation

**Figure 1:**
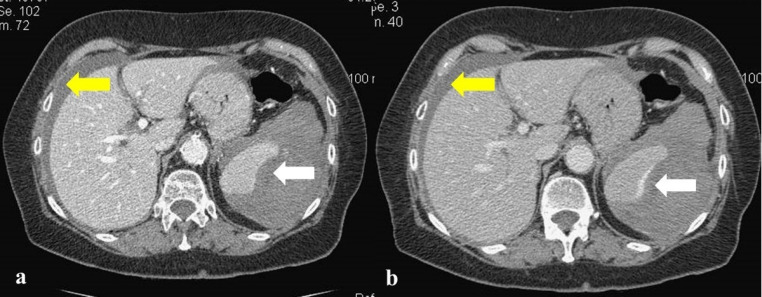
Abdominal computed tomography with intravenous contrast

A 75-year-old woman was admitted to the Emergency Department due to abdominal pain associated to a pre-syncopal condition. Twelve hours earlier, she had performed a complete colonoscopy as a post-polypectomy surveillance at another hospital. The patient reported an open-cholecystectomy, denied any kind of trauma in the previous days and mentioned that she was suffering from Hashimoto's thyroiditis and arterial hypertension, for which she was regularly in treatment with levothyroxine sodium and calcium channel blocker. No anticoagulant/antiplatelet agents were taken. On admission, the oral temperature was 38.6 °C, blood pressure 105/55 mmHg, pulse rate was 98/minute and the respiratory rate 17/minute. Increases in white blood cell count (16.4/mm^3^; neutrophils 85.1%) and plasma D-Dimer 359 (NR: 2-200 ng/ml) associated with anemia (hemoglobin 10.7 g/dl) were present. Renal, bilio-hepatic, and pancreatic functions were normal. Physical examination revealed signs of peritoneal reaction to palpation. At this point, it was decided to perform an abdominal focused assessment with sonography (FAST), which demonstrated a hemoperitoneum. A subsequent computed tomography (CT) with intravenous (IV) contrast highlighted, in addition to the hemoperitoneum (yellow arrows), the presence of a hematoma due to the rupture of the spleen (white arrow), as shown in Figure 1.

Abdominal CT scan revealed the presence of a splenic hematoma of about 11 cm (withe arrows) and a copious intra-abdominal hemoperitoneum with perihepatic fluid collection (yellow arrows), in both arterial (figure 1a) and portal phases (figure 1b). A diagnosis of grade IV splenic rupture was made, according to the AAST (American Association for the Surgery of Trauma) classification (5). The patient underwent an emergency laparotomy with splenectomy, recovered well, and was discharged ten days later in good condition. 

## Discussion

In a 2016 review, Jehangir A et al. identified 172 cases of splenic rupture and noted an incidence of 1–21/100.000 for this complication, pointing out that it had a higher predominance among females (71.5% of cases), with a mean age of 63 years, and a previous history of abdominal surgery in 50.8–65% of cases (6). In a recent structured review, which included 45 articles for a total of 68 patients, Ullah W et al. emphasized two very interesting points regarding this complication: 1) the main reasons for performing the colonoscopy were screening in 46% of cases and diagnostic in 28%; 2) complete splenic rupture was found in 22% of patients, whereas subcapsular hematoma, spleen laceration and spleen avulsion were present in 63% of them (7). As evidenced by the work of Chow BL et al., its real incidence could be underestimated both due to the non-reporting of cases and the non-publication of the same (8). Our patient, in addition to abdominal pain, manifested a presyncopal condition. In fact, hemodynamic instability was reported in 43.1% of patients known to be suffering from this disease (8). The diagnosis of a splenic rupture may be difficult, partly owing to the delayed onset of the symptoms, even partly because some physicians might not be aware of this rare complication. Early identification of this event is critical, as mortality rate is around 5% (9). As already affirmed above, we hold the view that Emergency doctors should take this endoscopic complication into account to make an early diagnosis and reduce the risk of mortality due to delayed treatment. Early recognition of this condition and the subsequent prompt treatment are essential to improve the outcome of these patients.

## Conclusion:

Splenic rupture may be a dangerous complication of colonoscopy. FAST abdominal sonography performed in the emergency room certainly represents an excellent first diagnostic examination in suspected cases. Nevertheless, abdominal CT scan represents the gold standard exam for detecting splenic rupture and splenectomy is required in most of cases. 
